# Using vital statistics to estimate the population-level impact of osteoporotic fractures on mortality based on death certificates, with an application to France (2000-2004)

**DOI:** 10.1186/1471-2458-9-344

**Published:** 2009-09-17

**Authors:** Nelly Ziadé, Eric Jougla, Joël Coste

**Affiliations:** 1 Research unit APEMAC, EA 4360, Nancy-Université, Université Paris-Descartes, Université Metz Paul Verlaine, Paris, France; 2 CépiDc, National Institute for Health and Medical Research, Le Vésinet, France; 3 Département de Médecine, Université Saint-Joseph, Faculté de Médecine, Beirut, Lebanon

## Abstract

**Background:**

We developed a methodology using vital statistics to estimate the impact of osteoporotic fractures on the mortality of an entire population, and applied it to France for the period 2000-2004.

**Methods:**

Current definitions of osteoporotic fractures were reviewed and their components identified. We used the International Classification of Diseases with national vital statistics data for the French adult population and performed cross-classifications between various components: age, sex, I-code (site) and E-code (mechanism of fracture). This methodology allowed identification of appropriate thresholds and categorization for each pertinent component.

**Results:**

2,625,743 death certificates were analyzed, 2.2% of which carried a mention of fracture. Hip fractures represented 55% of all deaths from fracture. Both sexes showed a similar pattern of mortality rates for all fracture sites, the rate increased with age from the age of 70 years. The E-high-energy code (present in 12% of death certificates with fractures) was found to be useful to rule-out non-osteoporotic fractures, and to correct the overestimation of mortality rates. Using this methodology, the crude number of deaths associated with fractures was estimated to be 57,753 and the number associated with osteoporotic fractures 46,849 (1.85% and 1.78% of all deaths, respectively).

**Conclusion:**

Osteoporotic fractures have a significant impact on overall population mortality.

## Background

Osteoporotic fractures are one of the leading causes of death in the elderly population [1] and make a major contribution to the overall burden of disease [2,3]. This burden is expected to increase further, with the increase in life expectancy [4,5] and, consequently in the incidence of fragility fractures, including minor fractures, which have been reported to be associated with greater mortality rates in the elderly [6].

Despite the growing incidence of osteoporotic fractures, there have been few studies addressing their impact on mortality in large populations. Most of the information available is from variously assembled cohorts of patients, followed up for limited periods of time, and assessing the mortality outcome after a fracture event [7-13].

Assessing the impact of osteoporotic fractures on the mortality of the entire population, i.e. how frequently osteoporotic fractures contribute to death in the population, is informative as concerns public health. The nationwide vital registration system is very useful for this purpose. Indeed, these systems are the only sources of data at the national level consistently available in most developed (and some developing) countries; they are exhaustive, low-cost, and allow international comparisons. This approach relies on the identification and analysis of the causes of death declared on the death certificate and coded according to the International Classification of Diseases (ICD), following specific rules established by the World Health Organization (WHO). Some causes of death, such as lung cancer or myocardial infarction, have simple and unequivocal ICD translations. Others, such as osteoporotic fractures, are more difficult to operationalize for several reasons, in particular because the underlying medical concept--osteoporosis--is poorly defined. Thus, operationalization and data analysis for osteoporotic fractures, using an internationally accepted coding system such as the ICD is a major challenge, and, as far as we are aware, has not been undertaken.

There have been attempts to translate complex medical concepts into one or a combination of ICD codes. For example, the National Center for Health Statistics (NCHS) proposed the use of cross-classifications between different categories of codes to capture the multidimensional aspects of injury-related mortality [14]. Nashold *et al*. used alcohol-related codes derived from different ICD chapters to capture alcohol-related mortality from US death certificates, using declarations of, for example, alcoholic cirrhosis and alcoholic psychosis, alcohol poisoning [15]. Another study by Armstrong *et al. *analyzed the category of ill-defined causes to quantify the contribution of sudden coronary heart disease to mortality [16]. Using a similar approach, and taking advantage of the richness of information describing injuries and fractures in death certificates, we describe a methodology to estimate the impact of osteoporotic fractures on the mortality of an entire population, and we apply this method to France, using vital statistics for the period 2000-2004.

## Methods

### Definitions and conceptual models

The internationally agreed definition of osteoporosis is "a progressive systemic skeletal disease characterized by low bone mass and micro-architectural deterioration of bone tissue with a consequent increase in bone fragility and susceptibility to fracture" [17].

Various operational definitions of osteoporotic fractures have been proposed. They are based on one or a combination of the following *components*: site of fracture, mechanism of occurrence, bone mineral density and demographic data, including age, race and sex (Table 1).

**Table 1:** Proposed operational definitions of osteoporotic fractures.

**Component (s)**	**Definition**	**Methodology**	**Reference**
Age	*Most *fractures in people aged more than 50 years are the result of osteoporosis	Expert opinion	[18]

Site	"Major" fractures linked to mortality in population-based studies: hip, vertebrae, pelvis, distal femur, proximal tibia, multiple ribs and proximal humerus	Prospective cohort study, Expert consensus based on literature search	[8] [46]

Mechanism	Fracture caused by injury that would be insufficient to fracture normal bone, i.e. fracture that occurs as a result of minimal trauma (low-energy trauma), such as a fall from standing height or less, or no identifiable trauma.	Expert consensus	[47]

Bone mineral density (BMD)	BMD value 2.5 standard deviation s(SD) or more below the mean for a young normal population of same sex and race (T-score), at the lumbar, hip or radius site	WHO report based on fracture risk assessment	[48]

Age, site, bone mineral density	Fractures occurring at a site associated with low BMD and which also increased in incidence after the age of 50 years	10-year fracture probability calculated from a large cohort	[21]

Age, sex, race, site	Differential probabilities of attribution to osteoporosis according to the combination of several variables	Expert consensus by the Delphi method	[26]

Each *component *included in the definitions has limitations preventing its use alone. The age cut-off of 50 years is widely accepted [18] but may have a very low specificity. Three major fracture sites are traditionally associated with osteoporosis (hip, vertebrae, and distal radius), but many others may also be considered osteoporotic [9,19-21]. Concerning the mechanism of occurrence, low-energy trauma is an intuitively accepted definition, although doubts have recently been raised about this notion [22]. Finally, bone mineral density is inversely correlated with fractures [23] but is an unsatisfactory marker, with low sensitivity and specificity [24]; moreover, this clinical tool cannot be used at the population level.

To improve the specificity of the operational definition of osteoporotic fracture, combinations of different components have been suggested (Table 1). For example, a gradient of fracture risk based on the combination of age, preferential sites and low BMD (bone mineral density) has been proposed [21,25]. Melton *et al*. estimated the probability of attributing a fracture to osteoporosis as a function of age, sex, race and site of fracture, using a Delphi method with osteoporosis experts [26].

### Operationalization of models

The above *components *can be operationalized, using available data from the vital statistics systems and WHO coding rules and ICD codes.

#### Information available and coded from the death certificate

The medical death certificate format recommended by the WHO, and used in many countries, consists of two parts. The first part is used for reporting the sequence of events leading to death, proceeding backwards from the final disease resulting in death, with the underlying cause stated last. The second part reports the contributing causes of death defined as "other significant conditions that contributed to death but did not lead to the underlying cause" [27].

The coding step involves attributing a digital code to each cause of death listed on the certificate, according to the latest revision of the International Classification of Diseases (ICD), and then selecting the underlying cause according to a dedicated set of rules.

Various aspects of the coding of injury-related deaths are relevant to our analysis. Two types of ICD codes are used to describe injuries: one that describes the nature (or site) of the injury (I-code, for example: hip fracture, injury of the head etc.), and one that describes the mechanism of the injury (E-code, for example: fall, motor vehicle crash etc.). As an ICD rule, the mechanism of injury, or E-code, is coded as the underlying cause-of-death and is the cause of death routinely published. The nature (site) of the injury is also recorded as an additional code. When the physician omits to note a traumatic event with a fracture diagnosis, the coding system automatically assigns a nonspecific E-code, X59 (accidental exposure to other and unspecified factors), unless a specific suitable underlying cause of death is available from the certificate.

#### Operationalization of the components from information in the death certificate

- Age: Age (available from all certificates) was divided into 5-year classes to test for any potential threshold; some definitions involve considering any fracture in the "elderly" as osteoporotic, so it was important to identify any age threshold at which a change of the pattern of fractures could be found.

- Sex: Sex (also available from all certificates) was considered for stratified analyses, as the pattern of fractures may differ between men and women.

- Race: Race was not considered in this study conducted in France, where the population is mostly (> 95 percent) Caucasian.

- Site of fracture [I-code]: The IMD (injury mortality diagnosis) matrix developed by the NCHS group was used to identify the codes of fractures (Table 2). This matrix organizes injury mortality data into meaningful groupings by body region and nature of injury [14].

**Table 2:** Operationalizations of the site and mechanism Components.

**Component**	**Classification**	**ICD-10 code**
	Hip fracture	S72.0, S72.1, S72.2, T93.1
	
	*Other major "osteoporotic" fractures *[7,39]	
	Vertebrae	S12.0-S12.9, S22.0, S22.1, S32.0, T08, T91.1
	Pelvis	S32.1-S32.5, S32.8
	Distal Femur	S72.4
Fracture site	Proximal Tibia	S82.1
	Ribs	S22.3, S22.4, S22.5
	Proximal Humerus	S42.2
	
(I-code)	Skull fracture	S02.0-S02.9, T90.2
	
	Multiple fractures	S32.7, T02.0-T02.9
	
	Others	S22.8, S22.9, S42.0, S42.1, S42.3-S42.9, S52.0-S52.9, S62.0-S62.8, S72.3, S72.7-S72.9, S82.0, S82.2-S82.9, S92.0-S92.9
		T92.1-T92.2, T93.2 (sequelae)
		T10, T12, T14.2 (unspecified region)
	
	Osteoporotic fracture/Site unspecified	M80.9

	*Low-energy:*	
	Fall on same level, fall while being carried or supported by other persons, fall involving wheelchair, bed, chair, other furniture, on and from stairs and steps	W01, W03, W04, W05, W06, W07, W08, W10, W18
	Unspecified fall	W19
	
Fracture mechanism	*High-energy:*	
	Intentional self-harm by jumping from a high place	X80
	Assault	Y01-Y04
	Aggressions	X85-Y09
(E-code)	Falling, jumping or pushed from a high place	Y30
	Transport accidents	V01-V99
	High-energy falls (ladder, scaffolding, building, tree, cliff, diving)	W11-17
	Exposure to external forces	W20-W64
	Intentional self-harm	X60-X84
	
	*Unspecified mechanism*	X59 (exposure to unspecified factor)

- Mechanism of occurrence [E-code]: Another matrix developed by the same NCHS group was used. This matrix classifies mechanisms of occurrence according to intention. We further divided the mechanisms into three categories: high-energy trauma, low-energy trauma and "unspecified mechanism" [28] (Table 2).

- Osteoporosis: The specific code used when an "osteoporotic fracture" is certified without mentioning the site of fracture (M809) was considered.

### Data analyzed

In France, death certification is mandatory, and must be performed by a medical doctor because burial requires a medical signature. Death certificates are exhaustive and data are available from 1968 onwards. The data are centralized, coded and analyzed at the CépiDc (Epidemiology Center for Medical Causes of Death). Quality control procedures are performed periodically [29,30] and several epidemiological research studies have been performed using these data [31-37].

Since the year 2000, the 10^th ^revision of the International Classification of Diseases (ICD-10) has been used in France, and the number of coded causes of death is exhaustive. The coding system is automated: 80% of death certificates are coded by software for automatic coding and attribution of the underlying cause. For reasons of international comparability, the knowledge base included in the French software uses the Mortality Medical Data System (MMDS) decision tables developed by the CDC-NCHS.

Here, we considered the period 2000 to 2004, during which around 2.7 million death certificates were registered in mainland France and were available for analysis. Only death certificates for people dying after the age of 20 years were included in our analysis. Note that in accordance with the ICD rule, the mechanism of fracture is automatically coded as the underlying cause of death, whereas the site of fracture is coded as an additional cause of death.

### Statistical methods

Crude death rates were calculated by age class (5-year periods from 20 to 95 years) and sex, using the corresponding demographic data provided by the National Institute for Statistics and Economic Studies (INSEE) as a source for estimating the denominator.

Cross-classifications between the four components of the definition of osteoporotic fractures available from the death certificate (age, sex, site and mechanism of fractures) were performed; the distribution of fractures according to these components was estimated to categorize fractures as "osteoporotic" or "other".

Age thresholds were identified using a graphical method based on the plotting of mortality rates on the y axis and age on the x axis and then fitting a straight line to the lowest mortality rate values. The first mortality rate above the fitted line indicates the age threshold for a "significant" increase in mortality rate. This method is used in factor analysis, under the name of Cattell's scree test [38]. High-energy/all fracture ratios were calculated for each type of fracture, and a threshold for a "high" ratio was identified using the same graphical method.

## Results

There were 2,658,805 death certificates registered in mainland France between 2000 and 2004; 2,625,743 of these death certificates concerned people who died after the age of 20 years and were included in our analysis.

Of these, 57,753 death certificates reported fracture (2.2%) and 31,459 specified the presence of a hip fracture (55% of all fractures and 1.2% of all death certificates).

The code "osteoporotic fracture" was found in 677 death certificates (0.03% of all death certificates and 1.2% of all fractures). This code was used in some death certificates with no indication of the specific site of fracture; we included this very small additional number of death certificates under the category "other fractures" of the variable "site of fracture".

Cross-classification between I-codes (site of fracture) and E-codes (mechanism of fractures) is shown in Table 3. Hip fracture was the most frequently recorded type of fracture on death certificates for both sexes (44% of all fractures in men, 61% of all fractures in women); the next most frequent were skull fractures for men (21%) and pelvis fractures for women (4%). The mechanism of injury (E-code) was not specified for 74% of deaths with fractures in men and 95% of deaths with fractures in women. "Low-energy" fractures made up 0.06% of all fractures in men and 0.08% of all fractures in women. The "High-energy" E-code was present on 26% of death certificates for men and 5% for women. The proportion of "High-energy" E-code also varied with fracture site, and two clear-cut patterns were identified according to the high-energy fracture/all fractures ratio: fractures involving the axial skeleton (vertebrae, pelvis, ribs, and skull) and multiple fractures--with the exception of pelvis fractures in women--were associated with a *higher ratio*; and those involving the hip and peripheral skeleton (distal femur, proximal tibia, proximal humerus)--with the exception of proximal tibia fractures in men--were associated with a *lower ratio*.

**Table 3:** Cross-classification between I-codes (site of fracture) and E-Codes (mechanism of fracture). Expressed as numbers of death certificates 2000-2004 (men, women).

	**E-code (mechanism of fracture)**									
	**Men (1,359,399 death certificates)**					**Women (1,299,406 death certificates)**				
	
**I-code**	**Low-energy**	**High-energy**	**Unspecified E-code^a^**	**High/All Ratio**	**Total (Men)**	**Low-energy**	**High-energy**	**Unspecified E-code^a^**	**High/All Ratio**	**Total (Women)**

Hip	6	36	9571	0.00	9613	8	19	21 819	0.00	21 846

**Peripheral Skeleton**										

Distal Humerus	0	1	117	0.01	118	0	2	357	0.01	359

Distal Femur	0	1	30	0.03	31	2	1	201	0.00	204

Proximal Tibia	0	5	34	0.13	39	0	1	78	0.01	79

**Axial Skeleton**										

Pelvis	0	161	626	0.20	787	0	79	1,403	0.05	1,482

Ribs	1	241	795	0.23	1,037	0	85	717	0.11	802

Vertebrae	1	736	792	0.48	1,529	2	229	719	0.24	950

**Others**										

Skull	3	3,301	1,245	0.73	4,549	5	606	533	0.53	1,144

Other fractures^b^	3	467	2,917	0.14	4,030	12	189	7,757	0.03	7,958

Multiple	0	463	289	0.62	752	1	229	857	0.21	1,087

**All fractures**	**14**	**5,412**	**16,416**	**0.25**	**21,842**	**30**	**1,438**	**34,443**	**0.04**	**35,911**

^a^Unspecified E-code refers to death certificates in which the underlying cause of death is given as "Exposure to unspecified factor (ICD10 code X59)" or in which no E-code is given.

^b^Other fractures: fractures at other sites (shafts of long bones, forearm, ankle, bones of hand and foot) and unspecified body region

**Figure 1:**
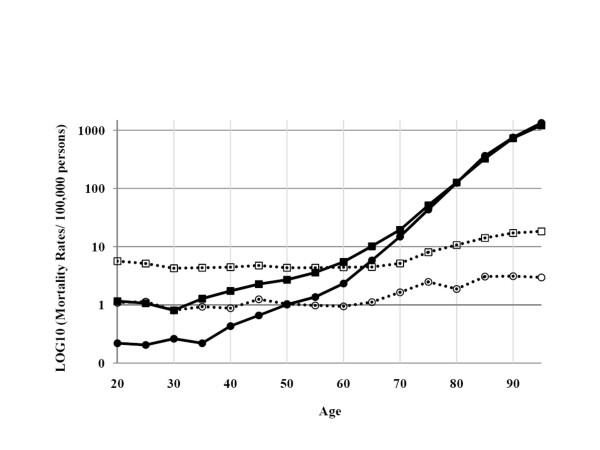
**Cross-classification between age and E-codes (Expressed as log10 (fracture-related mortality rates))**. Low+unspecified-energy mechanisms of fractures are shown in closed black squares for men, and closed black circles for women. High-energy fractures are shown in open squares for men and open circles for women.

**Figure 2:**
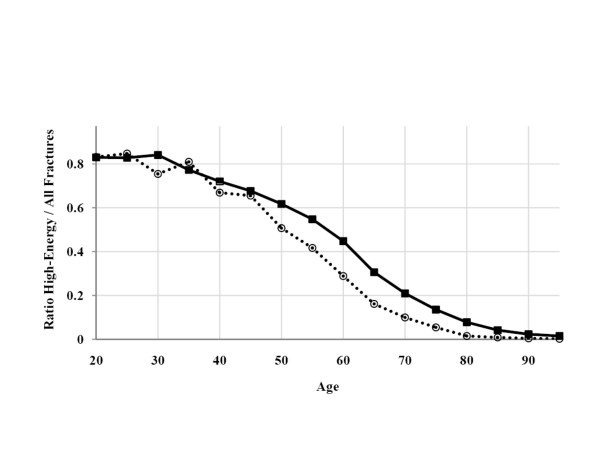
**The "high-energy fractures/all fractures" ratio by sex and age, using mortality rates/100,000 persons**. (Values for men are shown as closed black squares, and those for women are shown as open circles).

**Figure 3:**
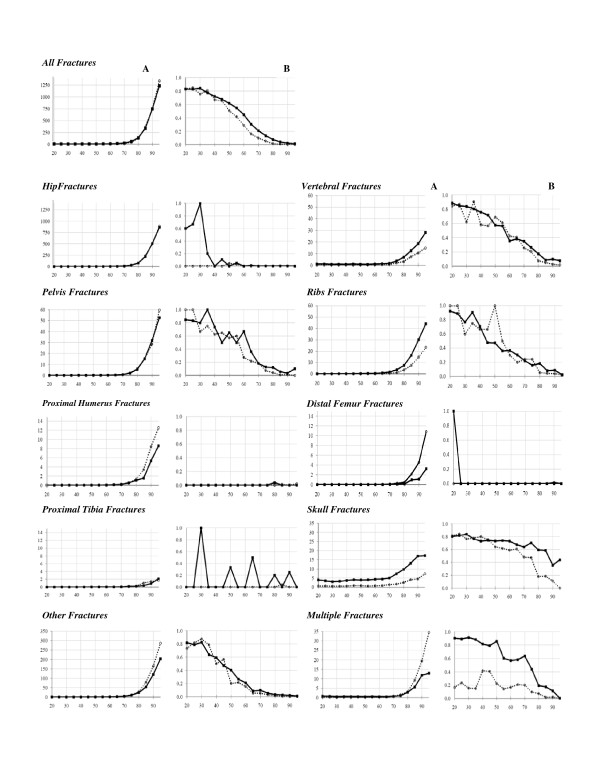
**Cross-classification between I-codes (site of fracture, expressed in mortality rates/100,000 persons) and age in years (Figure 3.A, first column) and between E-codes (mechanism of fracture, expressed by the "high-energy fractures/all fractures" ratio) and age in years (Figure 3.B, second column)**. Results are presented by sex and site of fracture. (Closed black squares for men and open circles for women). *NB. The scale of the vertical axis is not the same for all sites, due to large differences in fracture-associated mortality rates*.

Cross-classification between E-codes and age categories (Table 4) showed that low-energy E-codes (fracture related to fragility) was rare (almost entirely absent) until the age of 70 years. The high-energy code presented an interesting bi-modal pattern (Figure 1). High-energy fractures were frequent in younger age groups, especially in men, but also increased with age, suggesting a "fragility" component even when a high-energy trauma is certified. The high-energy codes/all fractures ratio remained constant with age (80% of fractures) until the age of 40 years (Figure 2), and decreased significantly from the age of 55 years in men and 45 years in women; these decreases for the two sexes were parallel. Figure 3-B present this ratio separately for each site of fracture. This pattern was found for each single fracture, except for skull and multiple fractures, for which the decrease started at an older age (60 years).

**Table 4:** Cross-classification between age and E-codes (mechanism of fracture). Expressed as mortality rates/100,000 persons (men, women. 2000-2004).

**Mortality rates/100,000 persons (death certificates 2000-2004)**								
	**Men**				**Women**			

**Age**	**Low-energy**	**High-energy**	**Unspecified E-code**	**High/All Ratio**	**Low-energy**	**High-energy**	**Unspecified E-code**	**High/All Ratio**

20-24	0.00	5.64	1.16	0.83	0.00	1.09	0.22	0.83

25-29	0.00	5.14	1.07	0.83	0.00	1.13	0.20	0.85

30-34	0.00	4.26	0.81	0.84	0.00	0.80	0.26	0.75

35-39	0.00	4.34	1.28	0.77	0.00	0.93	0.22	0.81

40-44	0.00	4.45	1.73	0.72	0.00	0.87	0.43	0.67

45-49	0.00	4.74	2.26	0.68	0.00	1.25	0.66	0.66

50-54	0.00	4.35	2.70	0.62	0.00	1.04	1.01	0.51

55-59	0.00	4.33	3.58	0.55	0.00	0.97	1.36	0.42

60-64	0.03	4.43	5.43	0.45	0.00	0.94	2.33	0.29

65-69	0.00	4.46	10.13	0.31	0.00	1.12	5.80	0.16

70-74	0.02	5.15	19.44	0.21	0.03	1.64	14.84	0.10

75-79	0.02	8.02	51.29	0.14	0.06	2.48	43.39	0.05

80-84	0.13	10.65	126.62	0.08	0.12	1.88	124.43	0.01

85-89	0.10	14.09	325.31	0.04	0.29	3.06	363.04	0.01

90-94	1.12	17.19	729.88	0.02	0.58	3.10	752.94	0.00

> = 95	1.08	18.29	1214.50	0.01	0.98	2.95	1334.40	0.00

Total	0.01	3.73	11.32	0.25	0.03	1.23	29.50	0.04

The cross-classification between I-codes and age categories is shown in Table 5 and Figure 3-A. All fractures increased with age, following a very similar pattern indicating a fragility component for all types of fractures, even for fractures not classically considered osteoporotic, such as skull fractures. An age threshold of 70 years was found for most fracture sites (highlighted in gray in Table 5). Note that hip fracture displays the same progression with age in men and in women, and that peripheral fractures are more common in women whereas axial fractures, especially skull fractures, are more common in men. The estimation of the number of deaths related to osteoporotic fractures should be corrected to exclude fractures due, in reality, to high-energy trauma. This fraction can be estimated by consideration of the (imperfect) sensitivity of the high-energy E-code, a consequence of certifiers failing to report all high-energy mechanisms of fracture on the death certificate. Indeed, for young adults (20-35 years), a group in which all fractures can be considered non osteoporotic, a high-energy code was recorded for only 80% of cases (considering all fractures together); the sensitivity of the E-code for identifying a "non osteoporotic" fracture can therefore be estimated at 80%. If we assume that the high-energy E-code is 100% specific (i.e. that this code is never recorded in cases of low-energy fracture), the reported fraction of fractures associated with a high-energy E-code (Figure 2) after the age of 70 years is presumably only 80% of the true value. Consequently, the reported value should be multiplied by 1.25 to estimate the true value for high-energy fracture. This corrected number can then be subtracted from the total number of fractures to obtain the number of osteoporotic fractures. Corrective factors were similarly computed for each site of fracture separately and were between 1.11 (multiple fractures in men) and 5 (multiple fractures in women).

**Table 5:** Cross-classification between I-codes and age (expressed as mortality rates/100,000 persons). Inflexion points are highlighted in bold.

**Age**	**Hip**	**Proximal Humerus**	**Proximal Tibia**	**Distal femur**	**Vertebrae**	**Ribs**	**Pelvis**	**Skull**	**Other Fractures^a^**	**Multiple Fractures**	**All fractures**
	**Men**										

20-24	0.05	0.00	0.00	0.01	1.07	0.13	0.13	3.93	0.74	0.74	6.80

25-29	0.03	0.00	0.00	0.00	1.07	0.09	0.18	3.51	0.67	0.65	6.20

30-34	0.01	0.00	0.01	0.00	0.81	0.12	0.09	3.05	0.53	0.43	5.07

35-39	0.05	0.01	0.00	0.01	0.90	0.19	0.10	3.28	0.54	0.55	5.62

40-44	0.04	0.00	0.00	0.00	0.86	0.23	0.18	3.70	0.66	0.51	6.18

45-49	0.18	0.01	0.01	0.00	1.02	0.22	0.14	4.07	0.86	0.50	7.01

50-54	0.44	0.01	0.03	0.01	0.84	0.37	0.19	3.93	0.80	0.41	7.04

55-59	0.73	0.01	0.04	0.00	0.79	0.45	0.17	4.02	1.13	0.53	7.91

60-64	1.75	0.03	0.05	0.00	1.02	0.47	0.23	4.34	1.52	0.44	9.90

65-69	4.35	0.11	0.03	0.00	1.32	0.92	0.41	4.46	2.45	0.39	14.59

**70-74**	**10.24**	0.17	0.04	**0.06**	**1.64**	**1.65**	**0.81**	5.04	**4.21**	**0.66**	**24.60**

75-79	30.33	**0.52**	**0.21**	0.09	3.54	3.73	2.22	**7.36**	9.94	0.97	59.34

80-84	78.75	1.09	0.21	0.13	6.96	7.54	5.87	9.77	23.18	2.85	137.39

85-89	220.06	1.54	0.39	0.96	12.45	16.40	15.24	13.02	52.00	5.60	339.49

90-94	511.14	5.36	0.89	1.12	18.53	30.13	31.92	16.96	115.84	11.83	748.19

> = 95	861.66	8.61	2.15	3.23	27.97	44.10	52.71	17.21	197.93	12.91	1233.86

Total	9.02	0.11	0.04	0.03	1.43	0.97	0.74	4.27	3.18	0.71	20.50
	**Women**										

20-24	0.01	0.00	0.00	0.00	0.27	0.02	0.03	0.63	0.63	0.19	1.31

25-29	0.01	0.00	0.00	0.00	0.23	0.01	0.04	0.64	0.64	0.17	1.34

30-34	0.01	0.00	0.00	0.00	0.20	0.05	0.03	0.51	0.51	0.12	1.07

35-39	0.01	0.00	0.00	0.00	0.19	0.07	0.04	0.53	0.53	0.18	1.15

40-44	0.07	0.00	0.00	0.00	0.29	0.06	0.07	0.60	0.60	0.11	1.31

45-49	0.11	0.01	0.01	0.00	0.22	0.06	0.16	0.87	0.87	0.25	1.91

50-54	0.22	0.01	0.00	0.00	0.28	0.03	0.07	0.85	0.85	0.17	2.04

55-59	0.39	0.01	0.00	0.01	0.34	0.07	0.06	0.57	0.57	0.26	2.34

60-64	0.75	0.01	0.01	0.01	0.38	0.15	0.16	0.75	0.75	0.27	3.27

65-69	2.44	0.11	0.01	0.03	0.52	0.21	0.32	0.86	0.86	0.34	6.92

**70-74**	**7.69**	**0.20**	**0.11**	0.09	0.67	0.58	**0.77**	1.40	**1.40**	**0.71**	**16.51**

75-79	25.21	0.46	0.06	**0.25**	**1.94**	**1.11**	1.92	1.72	1.72	1.48	45.93

80-84	78.24	1.32	0.29	0.46	3.20	3.56	5.20	**2.55**	2.55	3.03	126.43

85-89	236.64	3.40	1.05	2.18	7.34	7.59	15.44	4.07	4.07	9.27	366.40

90-94	499.72	8.45	1.44	4.55	10.69	14.80	28.59	4.55	4.55	19.42	756.62

> = 95	887.30	12.56	1.72	10.83	14.77	23.39	59.58	7.39	7.39	34.47	1338.33

Total	18.71	0.31	0.07	0.17	0.81	0.69	1.27	0.98	6.82	0.93	30.75

^a^Other fractures: fractures at other sites (shafts of long bones, forearm, ankle, bones of hand and foot) and unspecified body region

Using these correction factors and the age threshold for each site of fracture, we calculated the number of fractures that can be considered to have been osteoporotic: for each fracture site, the number of osteoporotic fractures was calculated as the total number of fractures recorded, starting from the corresponding age threshold, minus the number of high-energy fractures, multiplied by the sex- and site-specific correction factor (Table 6). The number of fractures attributed to osteoporosis was thereby estimated at 46,849 including skull fractures or 46,421 excluding skull fractures from the total estimate.

**Table 6:** Estimation of the impact of osteoporotic fractures on the mortality of the adult French population (death certificates after the age of 20 years, 2000-2004).

**Site**	**Number of Death Certificates With Fractures**	**Number of Death Certificates Above the Age Threshold^a^**	**Number of Death Certificates With High-Energy (E code) Fractures Above the Age Threshold**	**Correction factor^b^**	**Corrected Number of Death Certificates With High-Energy Fractures Above the Age Threshold Taking into Account the Imperfect Sensitivity of E-Code^c^**	**Corrected Number of Death Certificates With Fractures**
Hip	31,459	30,636	38	1.25	48	30,589

Pelvis	2,269	2,025	92	1.18	109	1,916

Ribs	1,839	1,473	148	1.14 (Men) 1.18 (Women)	170	1,303

Vertebrae	2,479	1,281	169	1.25 (Men) 1.28 (Women)	213	1,068

Multiple	1,839	1,167	106	1.11 (Men) 5 (Women)	258	909

Skull	5,693	1,068	514	1.25	643	425

Proximal Humerus	477	443	3	1.25	4	439

Distal Femur	235	222	1	1.25	1	221

Proximal Tibia	118	100	3	1.25	4	96

Other fractures^d^	11,345	10,099	174	1.25	218	9,882

TOTAL (without skull fractures)	52,060	47,446	734		1,025	46,421

TOTAL (with skull fractures)	57,753	48,514	1,248		1,668	46,846

^a^Age thresholds are identified for each site of fracture, based on the graphical method, see Figure 2A. (70 years for hip, pelvis, others, multiple, proximal humerus (women), proximal tibia (women), vertebrae (men) and ribs (men) fractures; 75 years for proximal humerus (men), proximal tibia (men), distal femur (women), vertebrae (women), ribs (women) and skull (men) fractures; 80 years for skull fractures (women)).

^b^The correction factors are identified for each site of fracture, using the estimation of the sensitivity of high-energy codes for identifying non osteoporotic fractures. They are derived from the high-energy fracture/all fracture ratio from death certificates for ages 20 to 35 years, an age group in which all fractures are considered non osteoporotic. For example, when the sensitivity of high-energy codes is 80% at a site, the number of fractures is inflated by 20% (multiplied by 1.25).

^c^Corrected number (rounded) = number of death certificates with high-energy (E-code) fractures above the age threshold * sex- and site-specific correction factor (1+(1-Sensitivity)).

^d^Other fractures: fractures at other sites (shafts of long bones, forearm, ankle, bones of hand and foot) and unspecified body region.

## Discussion

We developed a methodology to estimate the population-level impact of osteoporotic fractures on mortality, by developing operationally useful definitions of osteoporotic fractures and their components for demographic data and causes of death reported on death certificates. We found: 1) an age threshold at 70 years after which rates increased sharply for most fracture sites; 2) a similar pattern of rates for men and women; 3) similar patterns of rates for all sites of fracture, suggesting the involvement of all fractures, and not only those historically classified as "osteoporotic" fractures and 4) that the mechanism of injury was useful for the categorization of fractures, making it possible to exclude high-energy fractures by applying a correction factor. Using our methodology, we found that 46,849 deaths (1.78% of all deaths) in the adult French population between 2000 and 2004 were potentially related to an osteoporotic fracture (estimates of 46,421 and 1.76% were obtained if skull fractures were excluded from the total). This figure indicates a significant impact on general mortality. For example, during the same period in the same population, there were 16,600 deaths related to septicemia, 18,600 to renal failure and 51,300 to Alzheimer's disease.

The age threshold of 70 years appeared more relevant than that of 50 years conventionally used in practice for the definition of (clinical) osteoporotic fracture. This indicates that, from a mortality perspective, osteoporotic fractures have a significant impact on death from the age of 70 years onwards.

Some population-based cohort studies have reported excess mortality following osteoporotic fractures in women but not in men [39], whereas others [13] found increased hazard ratios for both men and women. Osteoporotic fractures are more prevalent in women, but the associated risk of death is higher in men, probably due to comorbidities. Our study indicates that the overall contribution of osteoporotic fractures to mortality is similar for men and women.

If we had chosen to limit our study to the generally accepted "major osteoporotic fracture sites", i.e. fractures of the hip, vertebrae, proximal humerus, distal femur, proximal tibia, pelvis and ribs, we would have underestimated the burden of osteoporotic fractures by 22%. We found that skull fractures occurring without a high-energy mechanism in older individuals represented 1.3% of all osteoporotic fractures recorded on death certificates. Although no association between this site and osteoporosis is classically reported [40], a review found that osteoporosis affected the bones of the skull [41] and some studies linked craniomaxillofacial fractures to osteoporosis [42-44] and to low-energy trauma in the elderly [45]. Our data suggest that skull fractures occurring after the age of 70 years and not associated with a high-energy code follow the same pattern as classical osteoporotic fractures, and therefore, may be included in the total estimate.

Concerning the mechanism of fractures, high-energy codes were found to be valuable for ruling out non osteoporotic fractures whereas low-energy codes did not bring significant additional information for the categorization of fractures as osteoporotic. We therefore suggest the exclusion of all high-energy fractures after 70 years of age (there are few) from the definition of osteoporotic fractures; furthermore the number of non osteoporotic fractures should be corrected to take into account the apparent poor sensitivity of the mention of high-energy codes in the certification process. In our study, this sensitivity was around 80% (from 20% to 90% according to the site), but it may vary between contexts, countries and times. The mechanism underlying many fractures was not specified on the death certificate and we assumed that these factures could be attributed to non significant i.e. low-energy traumatisms. However, investigations to characterize these unspecified mechanisms of injury would be useful to confirm this attribution.

The main strengths of our study are the exhaustive coverage of all French death certificates and the analysis of data over a five-year period, during which the same ICD (ICD-10) applied. We had full access to 2,658 805 death certificates, representing all deaths occurring in mainland France between 2000 and 2004. The data were consistent throughout the study period, and access to the original hand-written death certificates was possible (saved as scanned documents). The coding rules were uniform and transparent, and the selection of the underlying cause-of-death was unambiguous.

The study also has limitations, associated in particular with being based on death certificates. Various reasons for inaccuracies in death certificates arising at different stages of data generation and processing have been reported. Errors during death certification include ante mortem diagnosis errors, medical records being unavailable at the time of certification, lack of knowledge about the role of the fracture in death and, finally, misunderstanding of the certification process due to the inadequate training of doctors. However, our study does not aim to estimate the exact number of osteoporotic fractures in France or to assess mortality in the entire French population with osteoporotic fractures (or with osteoporosis, according to recent definitions), but attempts to identify cases in which the certifying physician considered the fracture to have played a significant role in death. Moreover, although for the cause of death is underreported for many chronic medical conditions, such underreporting is probably less frequent for injury-related deaths, for which the acute incident will generally attract the physician's attention.

Errors and inaccuracies may occur also at the stage of coding of the causes of death using the ICD. However, this is unlikely for fracture data, because there is no ambiguity in ICD codes for such injuries.

Another limitation concerns the determination of the age threshold for each fracture. The method we used was based on graphical estimation, and there is some subjectivity in the interpretations. However, the curves seem to have a clear cutoff, and a very similar threshold is found by analyzing all sites and both sexes; such consistency suggests that this approach is valid. Other data sources, such as health surveys, in-patient statistics and problem-specific medical registers, may provide more appropriate data for certain causes. However, mortality statistics are easier to obtain at the nationwide level. They remain the most comprehensive source of mortality statistics for the whole population, and cover long periods, thus facilitating the analysis of mortality trends over time. Our study describes a nationwide picture of acute osteoporotic fracture-related death, as judged by the physician filling in the death certificate; it is not a study of mortality in all patients previously diagnosed as having an osteoporotic fracture, nor does it establish how many people dying with an osteoporotic fracture actually had this diagnosis reported in their death certificate.

## Conclusion

In conclusion, we show that osteoporotic fractures have a substantial impact on general mortality. All sites showed a similar pattern of change with age, suggesting that fragility starts becoming a significant problem at the age of 70 years, for all fracture sites and for both sexes. Excluding the small proportion (about 6%) of high-energy fractures made the estimates more accurate. This methodological framework could be used in other international settings with similar death certification and coding systems for validation purposes. However, to improve further the categorization of the fractures, we recommend using the osteoporosis code more frequently, and, particularly, systematically using a specific mechanism of injury E-code. This will require greater awareness among certifying physicians concerning the significant impact of osteoporotic low-energy fractures on mortality.

## Abbreviations

BMD: Bone mineral density; CepiDc: Epidemiology Center for Medical Causes of Death; ICD-10: International Classification of Diseases, 10th version; MMDS: Mortality Medical Data System; NCHS: National Center for Health Statistics; WHO: World Health Organization.

## Competing interests

The authors declare that they have no competing interests.

## Authors' contributions

NZ performed the field activities (data collection, literature review) and statistical analysis, and wrote the manuscript. EJ helped supervise the field activities and participated in both designing the study and writing the manuscript. JC conceived the study, directed data analysis and contributed to writing the manuscript. All authors read and approved the final manuscript.

## Pre-publication history

The pre-publication history for this paper can be accessed here:


